# Dual extra-retinal origins of microglia in the model of retinal microglia repopulation

**DOI:** 10.1038/s41421-018-0011-8

**Published:** 2018-02-27

**Authors:** Yubin Huang, Zhen Xu, Shanshan Xiong, Guangrong Qin, Fangfang Sun, Jian Yang, Ti-Fei Yuan, Lei Zhao, Ke Wang, Yu-Xiang Liang, Lin Fu, Tianzhun Wu, Kwok-Fai So, Yanxia Rao, Bo Peng

**Affiliations:** 1 Shenzhen Institutes of Advanced Technology, Chinese Academy of Sciences, Shenzhen, Guangdong 518055 China; 2 Shanghai Center for Bioinformation Technology, Shanghai, 201203 China; 3 School of Biomedical Sciences, Li Ka Shing Faculty of Medicine, The University of Hong Kong, Hong Kong, China; 4 Shanghai Mental Health Center, Shanghai Jiao Tong University School of Medicine, Shanghai, 201108 China; 5 Guangdong-Hong Kong-Macau Institute of CNS Regeneration, Jinan University, Guangzhou, Guangdong 510632 China; 6 State Key Laboratory of Brain and Cognitive Sciences, The University of Hong Kong, Hong Kong, China; 7 Affiliated Eye Hospital, School of Ophthalmology and Optometry, Wenzhou Medical University, Wenzhou, Zhejiang 325000 China

## Abstract

Elucidating the origin of microglia is crucial for understanding their functions and homeostasis. Previous study has indicated that Nestin-positive progenitor cells differentiate into microglia and replenish the brain after depleting most brain microglia. Microglia have also shown the capacity to repopulate the retina after eliminating all retinal microglia. However, the origin(s) of repopulated retinal microglia is/are unknown. In this study, we aim to investigate the origins of repopulated microglia in the retina. Interestingly, we find that repopulated retinal microglia are not derived from Nestin-positive progenitor cells. Instead, they have two origins: the center-emerging microglia are derived from residual microglia in the optic nerve and the periphery-emerging microglia are derived from macrophages in the ciliary body/iris. Therefore, we have for the first time identified the extra-retinal origins of microglia in the adult mammalian retina by using a model of microglial repopulation, which may shed light on the target exploration of therapeutic interventions for retinal degenerative disorders.

## Introduction

Cell regeneration is relatively slow in the adult mammalian central nervous system (CNS)^1,2^. Though new neurons, astrocytes and oligodendrocytes can be generated during adulthood, neurogenesis and gliogenesis are relatively slow in the mammalian CNS due to the complicated regulations of intrinsic properties and extracellular environments^3–5^. Once the CNS get injured, the insulted cells cannot be repaired to the normal situation^4^. Thus, the consequences of CNS degeneration are permanent and irreversible, making it critical to understand the cell regeneration in CNS for regenerative medicine design.

Microglia are resident mononuclear phagocytes in the CNS, which play important roles in the development, homeostasis and diseases^6–10^. In the traditional understanding, there are no microglial progenitor cells in the CNS, and microglia maintain their population through self-renewal^11–13^. However, this notion has been challenged by a recent study that latent Nestin-positive cells in the brain are able to differentiate into microglia and repopulate the whole brain after pharmacological depletion of most resident microglia (~99%)^14,15^. As an important part of the CNS, the regenerative capacity is largely limited in the adult mammalian retina^3^. Although a few cells are sporadically regenerated in the retina^16–19^, no massive cell regeneration had been observed. Therefore, whether microglia can regenerate in the retina is unknown. If newly formed microglia can repopulate the whole retina after elimination of retinal microglia, it would be the first time observing a robust and massive cell regeneration in the retina. Furthermore, if repopulated retinal microglia are derived from the latent progenitor cell(s) as those in the brain, it will be the first definitive evidence that uncovers the progenitor or stem cell(s) in the adult mammalian retina. More importantly, it also provides insight into a novel target of therapeutic interventions for retinal degenerative disorders.

To this end, we first investigated the origin of repopulated microglia in the retina. New microglia were found to emerge and replenish the entire retina after elimination of all the resident microglia in the retina. Intriguingly, our fate mapping results demonstrated that Nestin-positive cells were not the precursor cells of repopulated retinal microglia, different from repopulated brain microglia. In contrast, repopulated retinal microglia have two populations of distinct origins: the majority of center-emerging microglia and the minority of periphery-emerging microglia. The center-emerging microglia were solely derived from residual microglia in the optic nerve, whereas the periphery-emerging microglia were derived from macrophages in the ciliary body/iris.

Therefore, by using the microglial repopulation model, we for the first time observed a robust and massive cell regeneration in the adult mammalian retina. Furthermore, we first identified extra-retinal origins of microglia in the adult retina. Our study sheds light on the origins and maintenance of microglia in the retina.

## Results

### Inhibition of CSF1R by PLX5622 completely eliminate retinal microglia

**Fig. 1:**
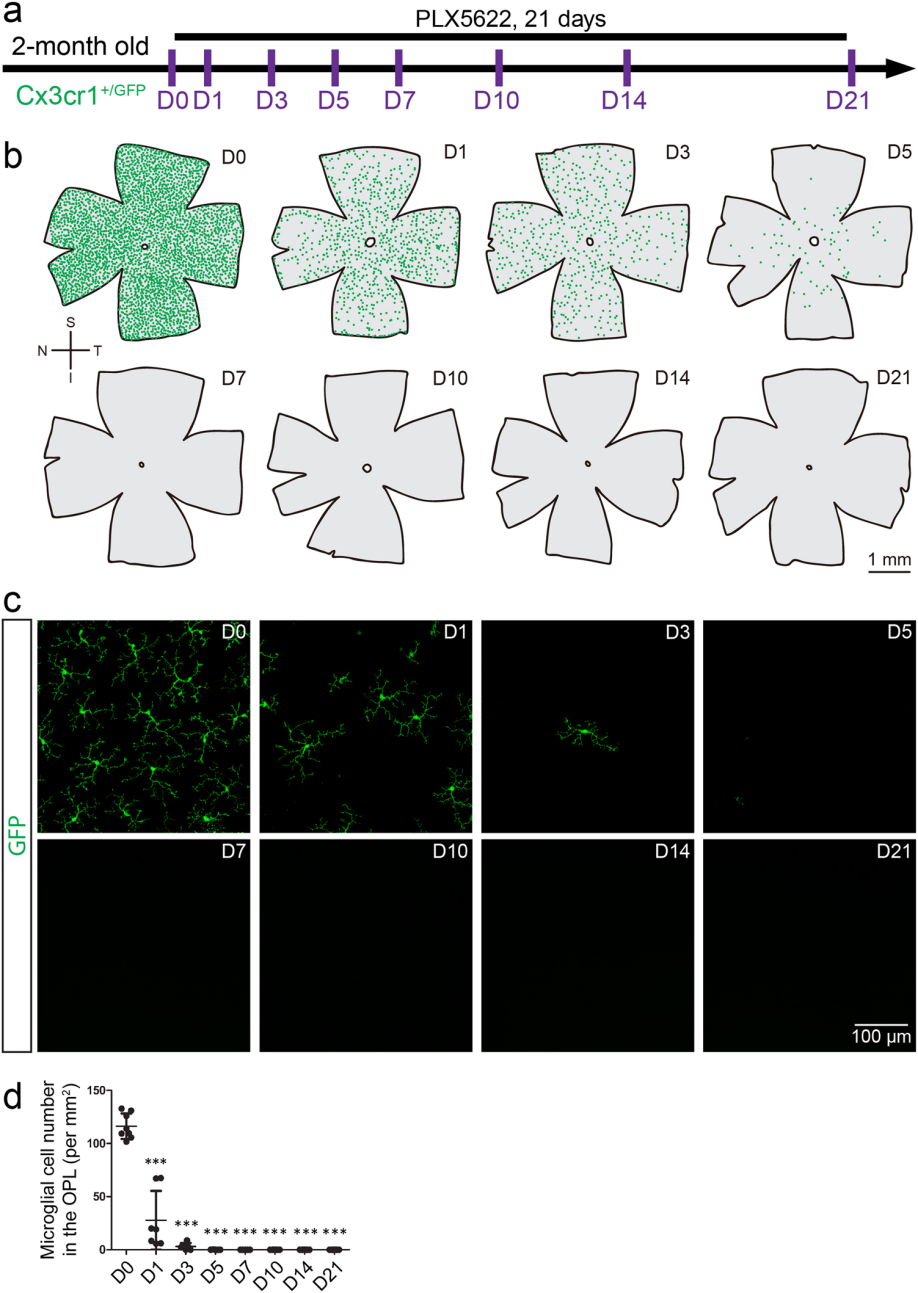
**Inhibition of CSF1R by PLX5622 rapidly depletes all microglia in the retina**. **a** Scheme of PLX5622 administration and time points for observation. **b** Spatial distributions of retinal microglia show that the cell numbers are reduced after PLX5622 administration. Each green dot represents a microglial cell. **c** Representative confocal images show microglial numbers are reduced in the OPL of the retina after PLX5622 administration. **d** Quantification of microglial density in the OPL of normal retinas and the retinas upon PLX5622 treatment. Green: GFP. S: superior; I: inferior; N: nasal; T: temporal; PLX5622: PLX5622 formulated diet. The data are presented as mean ± SD; *NS* not significant; *p < 0.05 to D0; **p < 0.01 to D0; ***p < 0.001 to D0. One-way ANOVA with Bonferroni’s *post hoc*

CSF1R is crucial for the viability of microglia. When CSF1R is genetically knocked out or pharmacologically inhibited, brain microglia are largely depleted^14,20,21^. Therefore, we utilized the selective CSF1R inhibitor PLX5622^22,23^ to deplete microglia in the retina. PLX5622 was formulated into a standard AIN-76A rodent diet (1200 ppm; Plexxikon). Two-month old Cx3cr1^+/GFP^ mice, in which all microglia express GFP^24^, were fed with PLX5622 formulated diet (PLX5622 for short) for up to 21 days (Fig. 1a). Twenty-four hours after CSF1R inhibition, microglial numbers were steeply reduced in both of the outer plexiform layer (OPL) (Figs. 1b-d; by 76.16%, *n* = 7, *p* < 0.001; *n* represents the number of retinas) and the inner plexiform layer (IPL) (Supplementary Figs. 1a-b; by 38.53%, *n* = 7, *p* < 0.001). Retinal microglia in the OPL were completely ablated after PLX5622 administration for 7 days and thereafter (Figs. 1b-d; 100%, *n* = 5-8, *p* < 0.001). Similarly, all microglia in the IPL were eliminated at day 10 of PLX5622 treatment and thereafter (Supplementary Figs. 1a-b; 100%, *n* = 8, *p* < 0.001). Notably, PLX5622 also depleted microglia in the brain (data not shown).

To further demonstrate whether retinal microglia are indeed removed instead of losing microglial markers (e.g., Cx3cr1) in response to PLX5622, we crossed Cx3cr1-CreER mice, in which all microglia express CreER^25–30^, with the tdTomato reporter line Ai14^31^ to obtain Cx3cr1-CreER::Ai14 mice for fate mapping. Neonatal Cx3cr1-CreER::Ai14 mice were administered with 50 μg tamoxifen intra-gastrically for 4 days to permanently label microglia (Supplementary Fig. 2a-b). At 2-month old, all microglia in the retina were labeled by tdTomato (*n* = 8). After PLX5622 administration for 5 days, the tdTomato-positive cell number in the retina markedly declined (Supplementary Fig. 2c-d), demonstrating the CSF1R inhibition resulted in *bona fide* depletion of microglia. Collectively, PLX5622 rapidly eliminates all retinal microglia within 10 days.

### Two distinct populations of microglia replenish the retina after withdrawal of the CSF1R inhibition

**Fig. 2:**
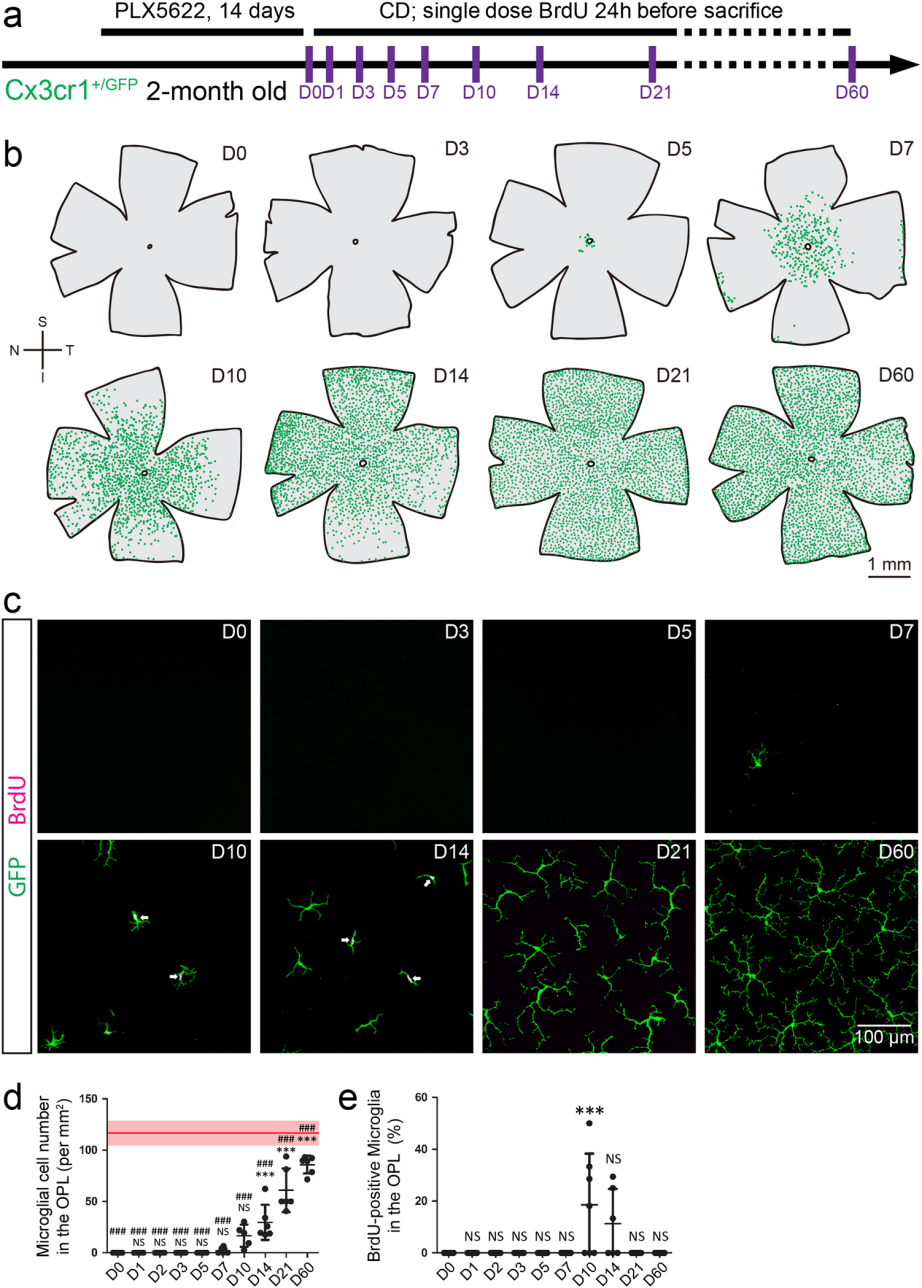
**Repopulated microglia replenish the entire retina after removal of CSF1R inhibition**. **a** Scheme of microglial repopulation and time points for examination. **b** Spatial distributions of retinal microglia show that microglia repopulate the whole retina after removal of PLX5622. Each green dot represents a microglial cell. **c** Zoom-in images of microglia in the OPL. Green: GFP; magenta: BrdU. **d** Quantification of microglial density in the OPL during microglial repopulation. The red line and pink area indicate the mean and SD of microglial density in normal retinas, respectively. *NS* not significant; *p < 0.05 to D0; **p < 0.01 to D0; ***p < 0.001 to D0; #*p* < 0.05 to the normal retina; ##*p* < 0.01 to the normal retina; ###*p* < 0.001 to the normal retina. **e** Quantification of BrdU-positive microglia among all microglia in the OPL during repopulation. *NS* not significant; *p < 0.05 to D0; **p < 0.01 to D0; ***p < 0.001 to D0. S: superior; I: inferior; N: nasal; T: temporal; PLX5622: PLX5622 formulated diet; CD: control diet. Arrows: BrdU + microglia. The data are presented as mean ± SD. One-way ANOVA with Bonferroni’s *post hoc*

Newly formed microglia are capable of replenishing the whole brain after ablation^14,32^. Due to the lack of progenitor/stem cells, no robust and massive cell regeneration has been observed in the adult mammalian retina^16^. Therefore, whether microglia can regenerate is largely unknown. To address this question, we first treated adult Cx3cr1^+/GFP^ mice with PLX5622 for 14 days to eliminate all retinal microglia. Then we replaced PLX5622 formulated diet by AIN-76A control diet (CD) (Fig. 2a). We found that repopulated microglia began to emerge in the central retina near the optic nerve head (ONH) at day 5 of recovery (Fig. 2b). Then center-emerging microglia primarily relocated in the retina in a center-to-periphery manner (Fig. 2b). Microglial cell numbers significantly increased in the OPL of the middle peripheral retina (50% eccentricity) at day 14-60 of repopulation (Figs. 2c, d; *n* = 6, *p* < 0.05 for day 14 and *p* < 0.001 for day 21 and 60), but the densities of repopulated microglia were still lower than resident microglia in naïve retinas (Fig. 2d; *n* = 6, *p* < 0.001). A similar but faster trend were observed in the IPL (Supplementary Fig. 3a-b; *n* = 5-8). By day 21 of repopulation, microglia in the IPL were recovered to the normal density (Supplementary Fig. 3a-b; *n* = 6, *p* > 0.05). In addition, BrdU incorporated microglia were detected in the retina from day 10 to day 14 of repopulation (Fig. 2e and Supplementary Fig. 3c; *n* = 6-8), indicating newly forming microglia underwent cell division (proliferation and/or differentiation).

In addition to the center-emerging microglia, a minority of repopulated microglia were observed emerging at the peripheral retina and spreading in a periphery-to-center manner. A small number of microglia sporadically appeared in the peripheral retina at day 7 of repopulation (7 out of 12 retinas) and became evident by day 10 (Fig. 2b). Out of 12 retinas, 5 were apparently demarcated by 2 populations of microglia at day 14 of repopulation (Supplementary Fig. 4a). The morphology of periphery-emerging microglia was less ramified than that of center-emerging cell (Supplementary Fig. 4b). The less ramified microglia can still be observed in the peripheral retina at day 60 of microglial repopulation (3 out of 6 retinas). These results suggest that, unlike the transient-lasting blood-borne microglia/macrophages found in the diseased CNS^33–35^, the periphery-emerging microglia in the retina are long-lasting cells in the retina. Converging evidence of distinct emerging sites, disparate distributions and diverse morphologies suggests that center and periphery-emerging microglia have different cellular origins. In spite of the differences, both populations underwent cell division in the repopulating retina (Supplementary Fig. 4b). Notably, the previous study has indicated that residual microglia in the brain could repopulate the brain through self-renewal^32^. In contrast, the residual retinal microglia did not contribute to microglial repopulation in the retina, since all retinal microglia were eliminated before repopulation (Figs. 1-2 and Supplementary Fig. 1-3).

Collectively, the majority of new microglia begin to emerge in the central retina and repopulate the microglia-free retina in a center-to-periphery manner, whereas a minority of new microglia initially emerge at the peripheral retina and repopulate the retina in a periphery-to-center manner. Although with different morphologies, both groups of microglia undergo cell division during repopulation.

### Repopulated retinal microglia are not derived from Nestin-positive cells

**Fig. 3:**
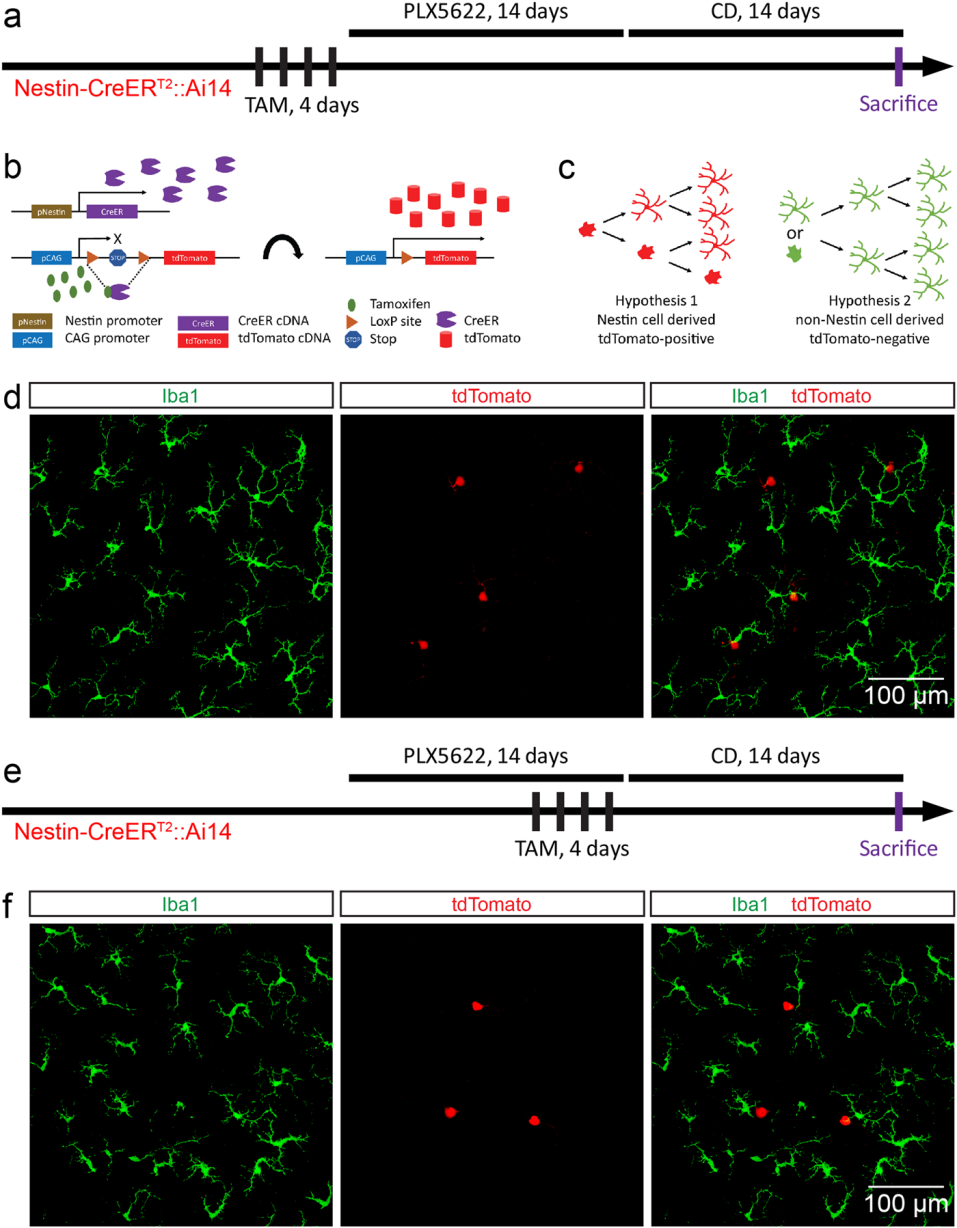
**Repopulated microglia of the retina are not derived from Nestin-positive cells**. **a** Scheme of tamoxifen triggered fate mapping for Nestin-expressing cells prior to the microglial depletion period. **b**, **c** The rationale and hypotheses of tamoxifen inducible fate mapping. **d** No tdTomato-positive microglia are found in the retina of the treatment protocol (a), in which tamoxifen is administered prior to microglial depletion. **e** Scheme of tamoxifen triggered fate mapping for Nestin-positive cells in the last 4 days of microglial depletion period. **f** No tdTomato-positive microglia are found in the retina of the treatment protocol (e), in which tamoxifen is administered during microglial depletion. S: superior; I: inferior; N: nasal; T: temporal; PLX5622: PLX5622 formulated diet; CD: control diet; TAM: tamoxifen. Green: Iba1; red: tdTomato

The previous study has indicated that repopulated brain microglia were primarily derived from Nestin-positive progenitor cells^14^. We thus asked whether microglial repopulation in the retina shared the same origin. To this end, we crossed the Nestin-CreER^T2^ mice, in which Nestin-positive cells express the tamoxifen inducible recombinase CreER^36^, with Ai14 mice for fate mapping. Adult Nestin-CreER^T2^::Ai14 mice were administered by tamoxifen (150 mg per kilogram of body weight) for 4 days to label Nestin-positive cells (Figs. 3a, b). Since tdTomato expression is induced by the irreversible genome recombination (Fig. 3b), Nestin-positive cells and their progenies permanently express tdTomato even if the cell fate changes. We reasoned if the newly formed microglia were derived from Nestin-positive cells, they must be labeled with tdTomato (Fig. 3c; hypothesis 1). Otherwise, the repopulated microglia should be tdTomato-negative (Fig. 3c; hypothesis 2). We thus removed all retinal microglia in tamoxifen-treated mice, followed by CD administration for 14 days (Fig. 3a). Surprisingly, tdTomato was not detected in repopulated retinal microglia (Fig. 3d; *n* = 10). There is an alternative possibility that the Nestin-positive progenitor cells could be a transient cell type that only emerges in response to the inhibition of CSF1R. To label the potential transient Nestin-positive progenitor cells (if any), we administered the animals by tamoxifen during the last 4 days of PLX5622 treatment (Fig. 3e). Still, we did not observe any tdTomato-positive microglia in the repopulated retina (Fig. 3f; *n* = 16). Therefore, the fate mapping results indicate that repopulated microglia of the retina are not originated from Nestin-positive cells, which is distinct from the repopulated brain microglia.

### Repopulated microglia in the retina are not derived from blood cells

Blood cells are able to differentiate into microglia in diseased retinas^33,37^. To identify whether repopulated retinal microglia are derived from blood cells, we first examined the integrity of the blood-retina-barrier (BRB) during microglial depletion and repopulation. Evans blue is a blood-brain-barrier (BBB) and BRB impermeable dye, which enters the CNS only when BBB/BRB are disrupted^38^. Mice of PLX5622 administered for 14 days and recovered for 7 days were intraperitoneally injected with Evans blue (2%, Sigma), respectively (Supplementary Fig. 5a). Evans blue was not detected in retinas or brains of either groups (Supplementary Fig. 5b-d; *N* = 5; *N* represents animal number), indicating the BRB and BBB remained intact during microglial depletion and repopulation.

Next, we examined whether blood cells gave rise to repopulated microglia by parabiosis as we previously described^33^. A beta-actin-GFP mouse, in which all cells are labeled by GFP^39^, was subcutaneously connected with a C57BL/6J wild-type (WT) mouse (Supplementary Fig. 5e). The blood cells exchanged between the parabionts. 14 days after the surgery, about half of blood cells in parabiotic WT mice were GFP-positive (Supplementary Fig. 5f-g; *N* = 5). Then we completely ablated retinal microglia of the WT-Cx3cr1^+/GFP^ parabionts by 14-day PLX5622 administration, followed by CD treatment for 21 days to allow microglial repopulation (Supplementary Fig. 5e). If blood cells were capable of giving rise to retinal microglia, GFP-positive microglia should be observed in repopulating retinas of WT parabiotic mice^33^. In contrast, we did not find GFP-positive microglia in all parabiotic WT retinas (Supplementary Fig. 5h-i; *n* = 14). Therefore, our data demonstrate that repopulated microglia are not derived from blood cells.

### Center-emerging repopulated microglia are derived from residual microglia in the optic nerve

Although retinal microglia were completely eliminated after 10 days of PLX5622 administration, some microglia in the optic nerve still survived after the CSF1R inhibition for up to 21 days (Supplementary Fig. 6; *n* = 8). Therefore, we hypothesized that surviving microglia in the optic nerve might migrate to the retina through the ONH, thus explaining the phenomenon of the center-emerging microglia.

**Fig. 4:**
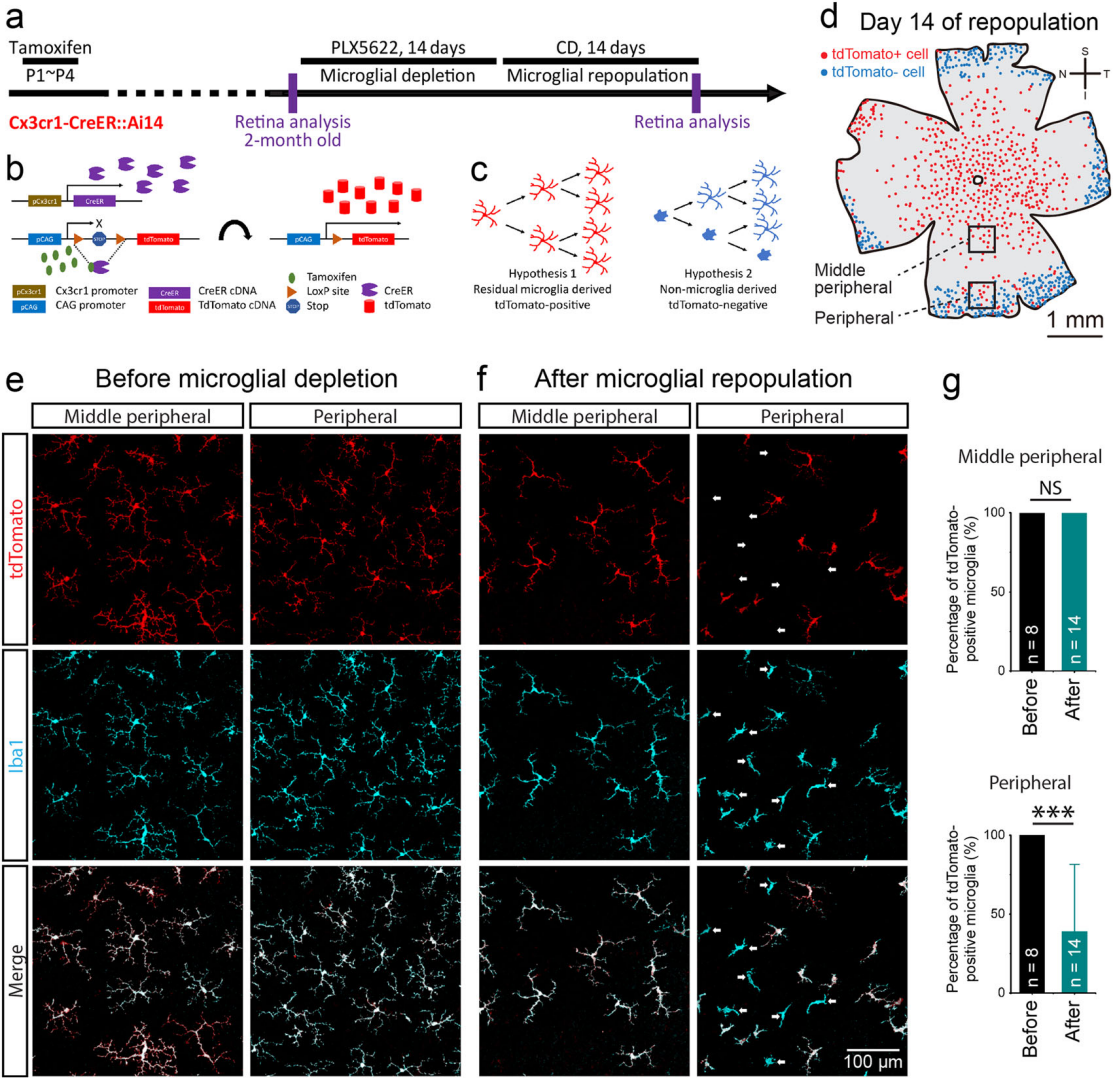
**Repopulated retinal microglia are primarily derived from residual microglia outside the retina**. **a** Scheme of genetic inducible fate mapping for resident microglia of the CNS. Tamoxifen was administered from P1 to P4. **b**, **c** The rationale and hypotheses of tamoxifen triggered fate mapping. **d** The representative spatial distribution of retinal microglia shows that there are tdTomato-positive and negative microglia in the retina 14 days after repopulation. Each red dot represents a tdTomato-positive microglial cell, while each cyan dot represents a tdTomato-negative microglial cell. **e** Confocal images show all retinal microglia of tamoxifen administered mice at 2-month old are labeled by tdTomato before microglial depletion. **f** Confocal images show all repopulated microglia in the middle peripheral retina are labeled by tdTomato, whereas some microglia in the peripheral retina are tdTomato-negative. Arrows: tdTomato-negative microglia.** g** Quantifications of tdTomato-positive cells in middle peripheral and peripheral retinas. S: superior; I: inferior; N: nasal; T: temporal; PLX5622: PLX5622 formulated diet; CD: control diet. Cyan: Iba1; red: tdTomato. The data are presented as mean ± SD. *NS* not significant; *p < 0.05; **p < 0.01; ***p < 0.001. Independent *t* test; *n* retina number for each group

To test this hypothesis, we first asked whether repopulated microglia were derived from residual microglia. We utilized Cx3cr1-CreER::Ai14 mice for fate mapping, in which all microglial cells are labeled after tamoxifen induction^25–27^. We administered tamoxifen in the neonatal mice (Fig. 4a). In the 2-month old mice, all microglia in the retina and optic nerve are labeled by tdTomato (Figs. 4e,g and Supplementary Fig. 6; *n* = 8). We did not find any tdTomato-positive non-microglial cells in the retina or optic nerve (data not shown). Therefore, tdTomato were exclusively expressed in all microglial cells, consistent with previous studies^26,40^. We then depleted all retinal microglia by PLX5622 for 14 days, followed by CD recovery for 14 days (Fig. 4a). If repopulated microglia are labeled by tdTomato, they must be derived from residual microglia (Fig. 4c; hypothesis 1). Otherwise, repopulated microglia are derived from other cells (Fig. 4c; hypothesis 2). We found that all repopulated microglia in the central and middle peripheral retina (center-emerging microglia) were tdTomato-positive (Figs. 4d, f, and g; *n* = 14, 100%). The results strongly suggest that the center-emerging repopulated microglia are solely derived from residual microglia in the optic nerve. Interestingly, a lot of repopulated microglia in the peripheral retina (periphery-emerging microglia) were tdTomato-negative (Figs. 4d, f, and g; arrows, *n* = 14, 61.04%), indicating a different origin from the center-emerging cells.

**Fig. 5:**
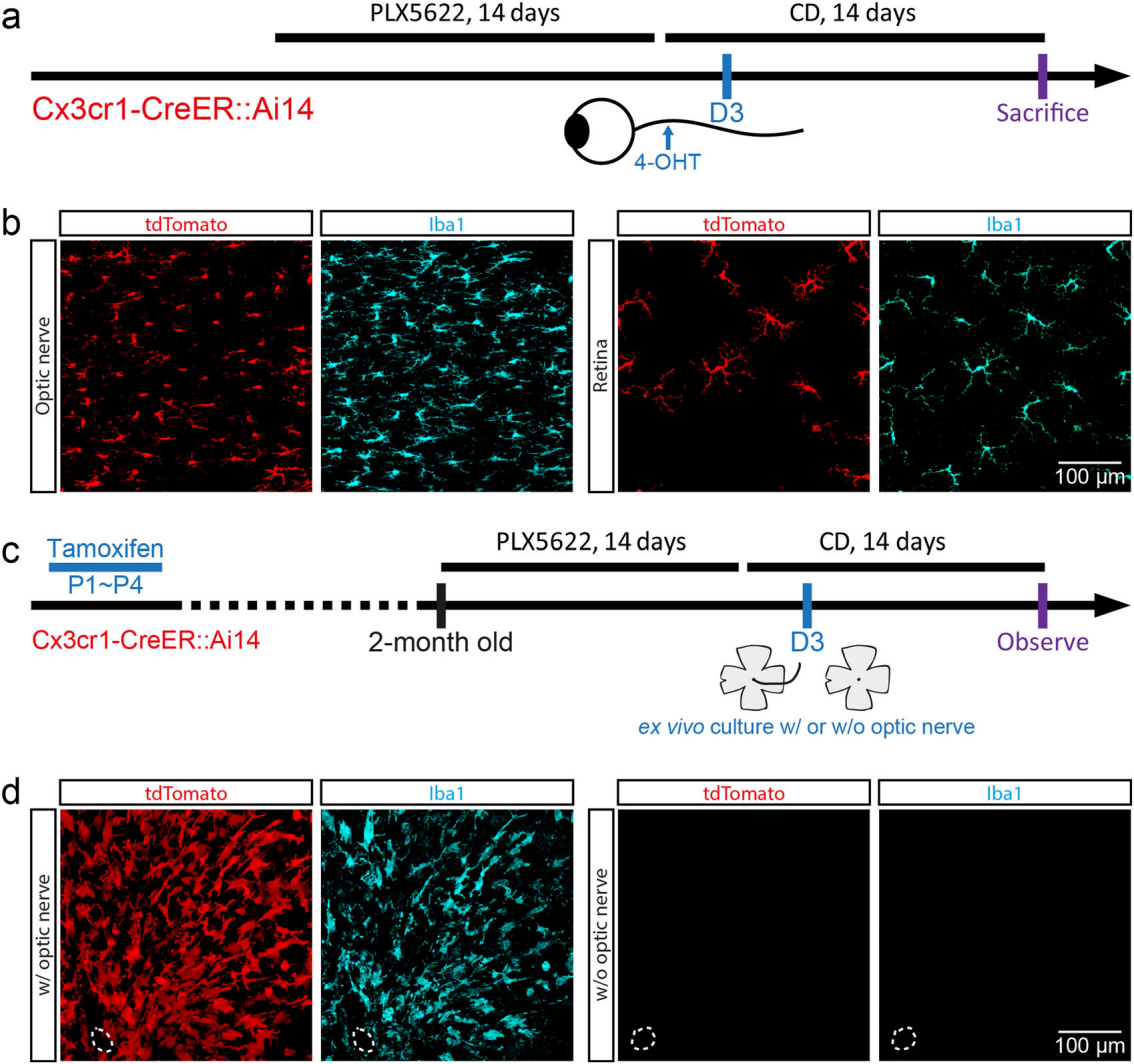
**Center-emerging microglia are derived from residual microglia in the optic nerve**. **a** Scheme of fate mapping for microglia in the optic nerve. **b** Confocal images show that the optic nerve microglia (left) and repopulated retinal microglia (right) express tdTomato in the retinas received intra-optic nerve injection of 4-OHT. **c** Scheme of ex vivo culture of retinal explants with or without the optic nerve. **d** Confocal images show that repopulated microglia emerge in the optic nerve-connected retinal explants after ex vivo culture for 11 days (left), but not in the optic nerve-removed retinal explants (right). PLX5622: PLX5622 formulated diet; CD: control diet. Cyan: Iba1; red: tdTomato. Dashed circle: optic nerve head

Next, we designed two experiments to investigate whether center-emerging microglia were derived from residual microglia in the optic nerve. First, we labeled microglia in the optic nerve by intra-optic nerve injection of 4-hydroxytamoxifen (4-OHT) (Fig. 5a). Some microglia in the optic nerve were labeled by tdTomato (Fig. 5b). We then applied the intra-optic nerve injection to label optic nerve microglia at day 3 of repopulation (Fig. 5a), when there were no microglia in the retina. At day 14 of repopulation, tdTomato-positive microglia were found in the middle peripheral retina (Fig. 5b; 10 out of 10 retinas). Notably, not all optic nerve microglia were labeled with tdTomato after one single dose of tamoxifen injection. Therefore, some repopulated retinal microglia were tdTomato-negative. The results indicate that the center-emerging microglia are derived from the residual microglia in the optic nerve. Second, we cultured microglia-free retinas from tamoxifen-administered Cx3cr1-CreER::Ai14 mice, with or without the optic nerve (Fig. 5c). Eleven days after ex vivo culture, repopulated microglia were found in the optic nerve-connected retinal explants, but not in the optic nerve-removed retinal explants (Fig. 5d; *n* = 6 and 12, respectively), indicating that residual microglia in the optic nerve contributed to center-emerging microglia. Collectively, our results by different approaches demonstrate that the center-emerging microglia are derived from the residual microglia in the optic nerve.

### Periphery-emerging microglia are derived from the ciliary body/iris macrophages

**Fig. 6:**
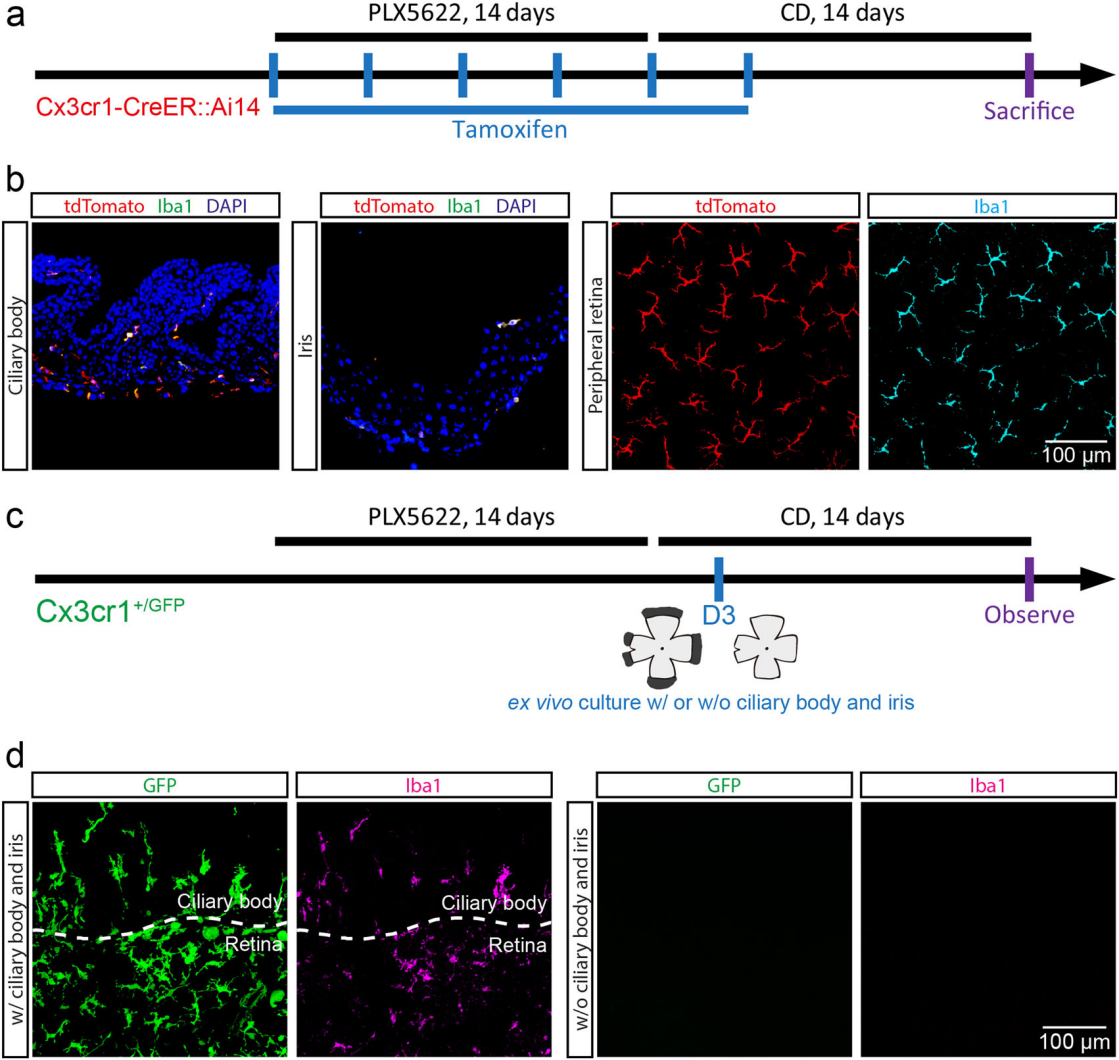
**Periphery-emerging microglia are derived from macrophages in the ciliary body/iris**. **a** Scheme of genetic inducible fate mapping for the ciliary body and iris macrophage derived microglia in repopulated retinas. **b** Confocal images show that all macrophages in the ciliary body/iris (left) and all repopulated microglia (right) in the peripheral retina are labeled with tdTomato. **c** Scheme of ex vivo culture of retinal explants with or without the ciliary body and iris. **d** Confocal images show that repopulated microglia emerge in the ciliary body and iris-connected retinal explants after ex vivo culture for 11 days (left), but not in the ciliary body and iris-removed retinal explants (right). PLX5622: PLX5622 formulated diet; CD: control diet. Cyan: Iba1; red: tdTomato; green: Iba1 (**b**) or GFP (**d**); magenta: Iba1; blue: DAPI

In contrast to the center-emerging microglia, some repopulated microglia in the peripheral retina were tdTomato-negative (Figs. 4d, f, and g; arrows; *n* = 14), suggesting a residual microglia-independent origin. In addition, these tdTomato-negative microglia exhibited a less ramified morphology (Figs. 4f-g), indicating they were the periphery-emerging cells. The evidence thus suggests that the periphery-emerging microglia are not derived from residual microglia. To determine the origin, we first examined the tdTomato expression pattern in macrophages of the ciliary body and iris, the affiliated tissues to the peripheral retina. In the adult Cx3cr1-CreER::Ai14 mice with neonatal tamoxifen treatment, some macrophages did not express tdTomato in the ciliary body and iris (Supplementary Fig. 7; arrow heads), probably due to the 2-month interval in the experimental procedure allowing the turnover of macrophages. We thus hypothesized that the periphery-emerging microglia were derived from ciliary body/iris macrophages. To test this hypothesis, we designed two experiments. First, we applied the tamoxifen administration from day 1 of depletion to day 4 of repopulation to minimize the number of tdTomato-negative macrophages (Fig. 6a). At day 4 of repopulation, when there were no microglia in the peripheral retina, all macrophages in the ciliary body and iris were labeled with tdTomato (Fig. 6b; *N* = 4). We then ceased the tamoxifen administration and examined the periphery-emerging microglia several days after repopulation (Fig. 6a). Distinct from the fate mapping results of the mice with two-month turnover interval, all periphery-emerging microglia expressed tdTomato in the mice without turnover interval (Fig. 6b; *N* = 4), indicating periphery-emerging microglia were derived from ciliary body/iris macrophages. Second, we cultured the microglia-free retinas with or without the ciliary body and iris (Fig. 6c). 11 days after ex vivo culture, new microglia appeared in the periphery of the ciliary body/iris-connected retinal explants (Fig. 6d; *n* = 5). In contrast, no microglia were found in ciliary body/iris-removed retinal explants (Fig. 6d; *n* = 5). Consequently, evidence from the genetic inducible fate mapping and ex vivo retinal explant culture indicates that the periphery-emerging microglia are derived from the ciliary body/iris macrophages.

### Repopulated microglia result in limited alterations of retinal transcriptomes

**Fig. 7:**
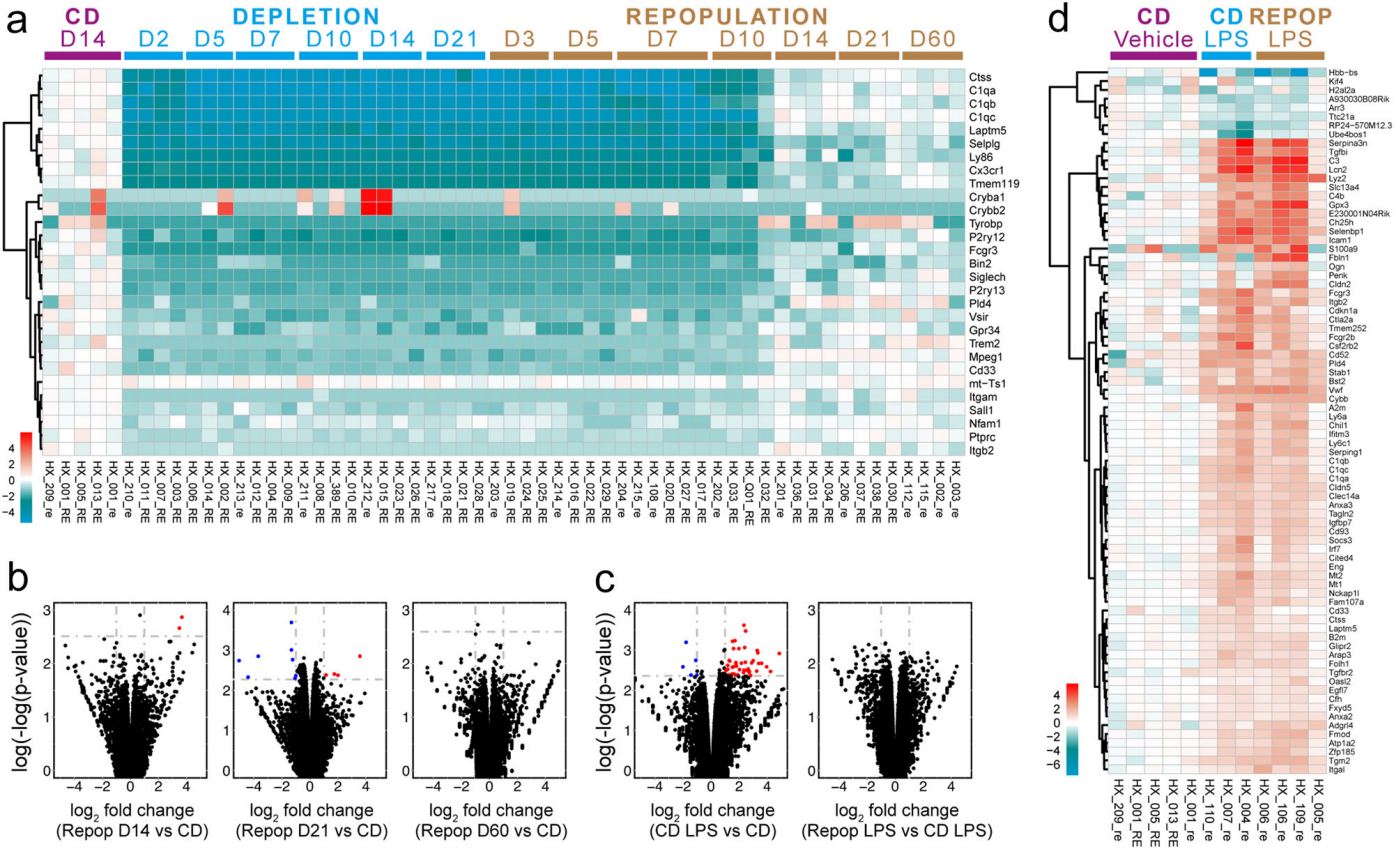
**The transcriptomes of the retina are little influenced by repopulated microglia**. **a** The heat map of retinal DEGs during microglial depletion and repopulation. **b** The volcano maps indicate the DEGs in microglial repopulated retinas compared to naïve retinas. **c** The volcano plots indicate the DEGs of the LPS challenged retinas. **d** The heat map of DEGs of the LPS challenged and vehicle treated retinas. CD: control diet, Repop: repopulation

As repopulated retinal microglia are originated outside the retina, whether their functions are similar to those of the resident microglia is completely unknown. To address this question, we analyzed the gene profiles of microglia-depleted, microglia-repopulated and normal retinas by RNA sequencing (RNA-Seq). During microglial depletion and repopulation, 29 genes were differentially expressed in the retina when compared with retinas of CD treated group (Fig. 7a). All differentially expressed genes (DEGs) during the depletion period were down-regulated, whereas most of them are known as microglia-related genes (e.g., *Cx3cr1*, *Itgam*, *Trem2*, *Sall1* and *Tmem119*) (Fig. 7a). No inflammation-related genes were up-regulated, indicating the microglial depletion did not induce the detrimental cytokine storm. During the first 10 days of microglial repopulation, the gene profiles were similar to those of microglia-free retina (Fig. 7a). Surprisingly, at day 14 of repopulation, only two genes were differentially expressed when compared to naïve retinas (Fig. 7b). The DEGs remained few in the retinas that repopulated for 21 days (Fig. 7b). At day 60 of repopulation, no genes were differentially expressed in repopulated retinas (Fig. 7b). The results indicate that the retina is little influenced by repopulated microglia, suggesting the similar function between resident and repopulated microglia in homeostasis.

Next, we asked whether repopulated microglia similarly responded to neuroinflammation as resident microglia. To this end, we systematically applied lipopolysaccharides (LPS; 1 mg/kg, Sigma) to activate microglia and then examined the gene profiles of the retina. The LPS challenge largely influenced the transcriptome of the retina with resident microglia, with 45 up-regulated and 5 down-regulated DEGs compared with those of naïve retinas (Fig. 7c). Interestingly, the transcriptome of the LPS-challenged mouse retina with repopulated microglia showed no significant differences from the LPS-challenged retina with resident microglia (Figs. 7c, d), suggesting repopulated and resident microglia shared similar characteristics in neuroinflammation. Therefore, our results implicate that the repopulated microglia may share similar functions as the resident microglia in homeostatic and diseased retinas.

**Fig. 8:**
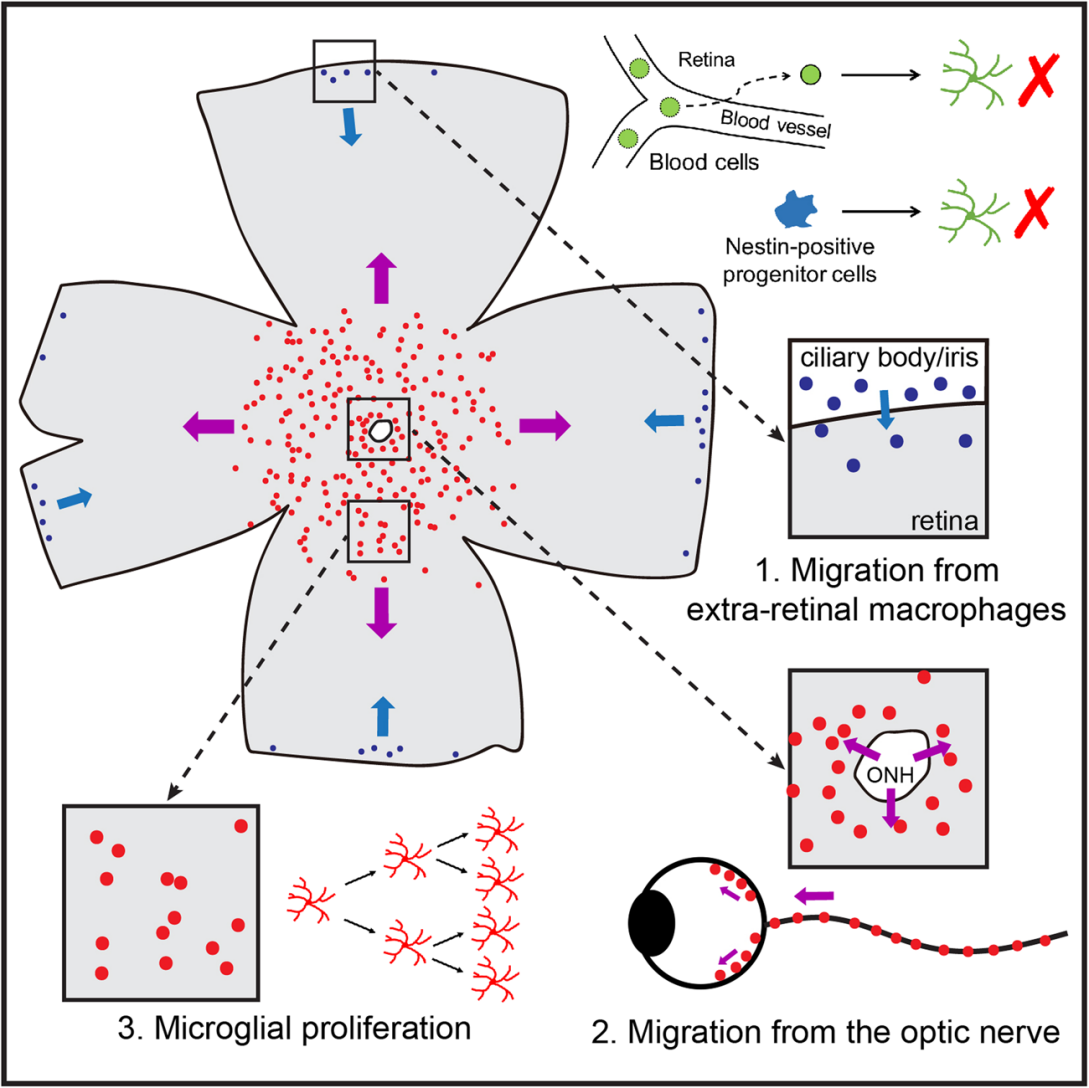
**Model of the extra-retinal origins of repopulated microglia in the retina**. Repopulated microglia are not derived from blood cells or Nestin-positive progenitor cells. Instead, they are derived from the ciliary body/iris macrophages (1) and the optic nerve microglia (2). Besides, repopulating microglia are undergoing proliferation (3)

Taken together, by eliminating retinal microglia, we have for the first time observed a robust and massive cell regeneration in the adult mammalian retina. The repopulated retinal microglia were not derived from Nestin-positive progenitor cells or blood cells (Fig. 8). Instead, the center-emerging microglia are derived from microglia in the optic nerve and the periphery-emerging microglia are derived from macrophages in the ciliary body/iris (Fig. 8). Besides, repopulating microglia are able to undergo proliferation (Fig. 8). Repopulated microglia may play similar roles as the resident microglia in heathy and diseased retinas.

## Discussion

### Microglial repopulation in the retina is not contributed by Nestin-positive cells or residual retinal microglia

In the previous study of the brain, endogenous Nestin-positive progenitor cells were suggested to be able to rapidly differentiate into microglia after 99% of brain microglia were depleted^14^. We found that microglia were able to regenerate in the microglia-free retina. We thus initially reasoned that the repopulated retinal microglia might share the similar origin of the brain. Unexpectedly, our fate mapping results reveal that the repopulated retinal microglia are not derived from Nestin-positive cells (Fig. 8), showing distinct cellular origins than the repopulated brain microglia. In brain repopulation study, microglia were not completely removed by pharmacological or genetic approaches^14^. Residual microglia also underwent proliferation during the repopulation period^14,32^. However, different from the brain, all microglia in the retina were depleted before the microglial repopulation. Thus, repopulated microglia cannot be derived from the residual microglia in the retina.

### Extra-retinal origins of retinal microglia

In the present study, we identified novel origins of retinal microglia by using the microglial repopulation model. Two distinct groups of extra-retinal cells contribute to repopulated microglia. First, the residual microglia in the optic nerve. They migrate to the retina through the optic nerve head. Then they repopulate the retina along the center-to-periphery axis (Fig. 8). This center-emerging population constitutes approximately 85% of the repopulated microglia. Second, the macrophages from the ciliary body/iris. The ciliary body is physically connected to the peripheral retina. Based on our results, macrophages in the ciliary body can relocate to the peripheral retina and migrate towards the central retina (Fig. 8). Since the ciliary body and iris are tightly connected, macrophages in the iris are possible to migrate to the peripheral retina through the ciliary body (Fig. 8). This periphery-emerging population accounts for around 15% of the repopulated microglia, and exhibits obvious differences in morphology. The morphologies of microglia have been believed to be primarily determined by the microenvironment^9,10,41^. Based on our observation, microglial morphologies may be also determined by intrinsic properties of the cell per se.

The ontogeny of CNS microglia and peripheral macrophages had been hotly debated for decades. For a long time, the homeostasis of microglia and macrophages had been thought to be maintained by replacement of myeloid cells in the blood^9^. However, researchers demonstrated that microglia and macrophages are autonomous and independent populations maintained by self-renewal^9,25,42–44^. Also most of non-microglia macrophages in the brain, except for choroid plexus macrophages, established stable populations^29^. Even under pathological conditions, the contribution of blood cells is minimal, if any^9,33–35,45^. In this study, we for the first time uncovered the peripheral macrophage-derived microglia. Their less ramified morphology suggests distinct intrinsic properties of this population, though the functional implications are unknown.

### Radial migration routes of retinal microglia

In the present study, our results also indicate novel migration routes of retinal microglia. In the diseased retina, the vertical migration of microglia has been observed. For instance, insulted photoreceptors in retinitis pigmentosa attracts microglia in the inner retina to migrate towards the outer retina^41,46^. However, the radial migration of microglia (either centrifugally or centripetally) has not been reported in the retina, especially for the long-distance migration. In our study, the center-emerging microglia migrated along the center-to-periphery axis and the periphery-emerging microglia migrated along the periphery-to-center axis. The physiological and pathological roles of the microglial radial migration need to be further elucidated. Furthermore, what signals attract microglia to radially migrate in the retina is unknown. Microglial migration in pathological conditions is triggered by inflammatory signals. However, based on our study, inflammatory response was not detected during the microglial depletion or repopulation period. Therefore, other factors may direct the microglia to migrate. Investigating the underlying mechanism of microglial migration in repopulation will provide new insights on understanding the regulatory principles of microglia.

## Materials and methods

### Ethics statements

All animal experiments were performed in accordance with the guidelines of the Institutional Animal Care and Use Committee (IACUC) at Shenzhen Institutes of Advanced Technology, Chinese Academy of Sciences.

### Animals

C57BL/6J, beta-actin-GFP (C57BL/6-Tg(CAG-EGFP)131Osb/LeySopJ, JAX: 006567), Nestin-CreER^T2^ (C57BL/6-Tg(Nes-cre/ERT2)KEisc/J, JAX: 016261), Cx3cr1^GFP/GFP^ (B6.129P-Cx3cr1tm1Litt/J, JAX: 005582), Cx3cr1-CreER (B6.129P2(C)-Cx3cr1tm2.1(cre/ERT2)Jung/J, JAX: 020940) and Ai14 (B6.Cg-Gt(ROSA)26Sortm14(CAG-tdTomato)Hze/J, JAX: 007914) mice were purchased from The Jackson Laboratories. Cx3cr1^+/GFP^ knock-in mice were obtained by crossing Cx3cr1^GFP/GFP^ with C57BL/6J mice. Ai14 mice were crossed with Nestin-CreER^T2^ and Cx3cr1-CreER mice to obtain Nestin-CreER^T2^::Ai14 and Cx3cr1-CreER::Ai14 mice, respectively. All mice were housed in the laboratory animal facility on a 12 h light and 12 h dark cycle with food and water ad libitum.

### Drug administration

To deplete retinal microglia, mice were fed with PLX5622 formulated AIN-76A diet (1200 ppm, Plexxikon) at libitum. The control animals were fed with normal AIN-76A diet (Plexxikon). To systematically induce CreER dependent recombination, mice were intraperitoneally administered with tamoxifen (150 mg per kg of body weight, Sigma, C8267) dissolved in corn oil for consecutive several days^31^. For the neonatal mouse, 50 μg tamoxifen was intra-gastrically injected for consecutive 4 days as previously described^47,48^. To locally induce CreER dependent recombination, Cx3cr1-CreER::Ai14 mice were administered with 4-hydroxytamoxifen (4-OHT, Sigma, T176) by intra-optic nerve injection (2 mm to the eye ball, 0.5 μL, 5 mg/mL in 50% DMSO).

### Evans blue assay

To measure the BRB and BBB integrity during the periods of microglial depletion and repopulation, 2-month old male mice were fed by PLX5622 or CD for 14 days, and PLX5622 for 14 days followed by CD for 7 days. Mice were then intraperitoneally injected with 0.2 mL 2% Evans blue (Sigma). Six hours after injection, mice were systematically perfused by 0.9% normal saline and tissues were then harvested. Tissue homogenate was incubated with formamide (1 g tissue in 10 mL formamide, Sigma) at 55 °C for 24 h to extract Evans blue. After that, the formamide-Evans blue mixture was centrifuged and the supernatant absorbance was measured at 610 nm by a microplate reader.

### Parabiosis surgery

The parabiosis was performed following the procedures we previously described with minimal modifications^33^. In brief, one female C57BL/6J mouse was co-housed with one beta-act-GFP or Cx3cr1^+/GFP^ mouse of the similar size and body weight for at least 2 weeks before the surgery to ensure the cohabitation of harmony. After deeply anesthetized with chloral hydrate (500 mg/kg, Sigma 15307), the opposite flanks of two mice were shaved and cleaned by the ethanol and Betadine solution from the elbow to the knee, before surgical incisions were made. Next, the skins were gently detached from the subcutaneous fascia to expose around 0.5 cm of free skin. After that, the adjacent elbow and knee joints were connected by the non-absorbable 3–0 suture and sutured the skins of two animals with 5–0 absorbable sutures (Ethicon, NJ). Erythromycin ointment was applied to the sutured area. Each mouse was then administered with 0.5 mL normal saline by intraperitoneally injection after procedures. Body temperatures were maintained at 37 ℃ by a heating pad until recovery. Meloxicam (1 mg per kg body weight, Sigma) was applied to limit infection and relieve pain. Sulfamethoxazole (2 mg/mL) and Trimethoprim (0.4 mg/mL) were added in the drinking water for 10 days to prevent potential bacterial infections. The WT/beta-actin-GFP parabiotic partners were used for blood cell analysis and the WT/Cx3cr1^+/GFP^ parabiotic partners were used to study the blood-borne origin of microglia.

### Flow cytometry

The whole blood was harvested in 50 mM EDTA (Sigma) in Dulbecco’s Phosphate-Buffered Saline (DPBS, HyClone, SH30028.02). The cells were incubated in ACK buffer at room temperature (Life Technologies, A10492-01) for 5 min to remove erythrocytes, followed by aspirating the supernatant and resuspending the pellets with FACS buffer (0.5% BSA and 2 mM EDTA in DPBS). After rinsed by FACS buffer for 3 changes, the cells were incubated with 7-AAD (1:80, BD Pharmingen, 559925) on ice for 10 min to label dead cells. Then the cells were analyzed by BD FACS Canto II (BD Biosciences). The blood chimera was identified by the percentage of GFP-positive blood cells in the parabiotic C57BL/6J mice. The cytometric flow data were then analyzed and plotted by FlowJo 7.6.1.

### Tissue preparation

Animals were deeply anesthetized by chloral hydrate (500 mg/kg body weight). For histological experiments, animals were perfused by 0.01 M PBS (Sigma, P4417) and 4% paraformaldehyde (PFA) (Sigma, 441244), respectively. Retinas and optic nerves were then collected and post-fixed in 4% PFA at 4 ℃ for 2 h. For the RNA-Seq, mouse retinas were immediately harvested in cold DPBS after deep anesthetization. Then retinas were quickly frozen in liquid nitrogen, and stored at −80 ℃ until further processes.

### Cryosection preparation

The ciliary body and iris were dehydrated in 30% sucrose in 0.01 M PBS at 4 ℃ for 2 days. After embedded in optimal cutting temperature compound (OCT, Tissue-Tek), the ciliary body and iris were quickly frozen by liquid nitrogen and then stored at −80 ℃ before sectioning. The ciliary body and iris with regions of interest (ROIs) were sectioned by the cryostat (Leica, CM1950) at the thickness of 20 μm.

### Immunohistochemistry

Whole-mount retinas and optic nerves were rinsed by PBS 10 min for 3 times, followed by 4% normal goat serum (NGS, Jackson Immuno, 005-000-121) or normal donkey serum (NDS, Jackson Immuno, 017-000-121) in 0.01 M PBS containing 0.5% Triton X-100 (Sigma-Aldrich T8787) (PBST) blocking at room temperature (RT) for 2 h. Then the samples were incubated with primary antibodies in 1% NGS or NDS in PBST at 4 ℃ overnight. After rinsed by PBST for 3 changes, samples were incubated with Alexa Fluor dyes conjugated secondary antibodies in 1% NGS or NDS in PBST with 4′,6-diamidino-2-phenylindole (DAPI, 1:1000, Sigma-Aldrich, D9542) at RT for 2 h. After that, samples were well rinsed for 3 times before mounted with anti-fade mounting medium (Vectashield H-1000).

Primary antibodies included rabbit anti-Iba1 (1:500, Wako, 019-19741), rat anti-BrdU (1:200, Abcam, AB6326), rabbit anti-GFP (1:500, Invitrogen, A11122), chicken anti-GFP (1:200, Millipore, AB16901) and goat anti-mCherry (1:500, Biorbyt, orb11618). Secondary antibodies conjugated to Alexa Fluor 488, Alexa Fluor 568 and Alexa Fluor 647 (Molecular Probes) were 1:500 diluted.

### BrdU incorporation assay

A single dose of BrdU (50 mg/kg, Invitrogen, 00-0103) was administered to the animals 24 h prior to sacrifice. Whole-mount retinas were denatured by 2 M hydrochloric acid for 20 min at RT, followed by renaturation with 0.01 M borate buffer (pH 8.2, Sigma-Aldrich 08059) at RT for 30 min before 0.01 M PBS rinse for 10 min. The immunohistochemistry was then carried out afterwards.

### Retinal explant ex vivo culture

Retinas from Cx3cr1-CreER::Ai14 or Cx3cr1^+/GFP^ mice were quickly dissected in the 95% O_2_ and 5% CO_2_ bubbled Ames Medium (Sigma). Then the retinal explant was carefully flattened on a millicell insert (Millipore, PICM03050) in the ganglion cell-up manner. After that, the retinal explant was transferred to the six-well plate. The retinal explants were cultured in Ames Medium with 20% heat inactivated horse serum (Gibco), 16 ng/mL colony stimulating factor 1 (Sigma), 10 ng/mL IL-34 (Sigma), and 1:100 penicillin-streptomycin (Invitrogen). The retinal explants were placed in the air-medium interface and maintained at 37 ℃ in a humidified atmosphere with 5% CO_2_. The culture medium was changed every 2 days.

### Microscopy

Confocal Images were taken by solid state lasers equipped Carl Zeiss LSM 700 confocal microscope. Plan-Apochromat 20 × (0.8 NA) 40 × (1.4 NA, Oil) or 63 × (1.4 NA, Oil) objectives were used. *Z*-stacked focal plans were taken and then maximal projected by ZEN 2.1 (Carl Zeiss). Images were adjusted by ZEN 2.1 (Carl Zeiss) and/or ImageJ (NIH) if necessary. Images were taken in the middle peripheral retina (50% eccentricity) unless specified.

Fluorescence images of the whole retina were acquired by the Nikon TI-U microscope equipped with a motorized stage (Nikon TI-S-E). Whole retina images were merged automatically by either Nikon NIS-Elements V4.50 (Nikon) or Photoshop CS3 (Adobe). Images were adjusted by ZEN 2.1 (Carl Zeiss) and/or ImageJ (NIH) if necessary.

### Data analysis

Microglial cell numbers were counted by Nikon TI-U microscope. Microglia were visualized by GFP, tdTomato or Iba1. Sampling areas were 10×, 20×, or 40× images at ROIs for each animal.

### RNA sequencing

Total RNA was extracted from retinal homogenate using the TriPure Isolation Reagent (Roche) following the manufacturer’s protocol. Then the RNAs were sent to BGI for 50 bp single-end sequencing by BGISEQ-500 sequencer. In Brief, Oligo (dT) magnetic beads were first used to select mRNA with polyA tail, or hybridized the rRNA with DNA probe and digested the DNA/RNA hybrid strand, followed by DNase I reaction to remove DNA probe. After the target RNA were obtained after purification, they were fragmented and reverse transcripted to the double-strand cDNA (dscDNA) by N6 random primer. Then the dscDNA was end repaired with phosphate at 5′ end and stickiness “A” at 3′ end, then ligated and adapted with stickiness T at 3′ end to the dscDNA. Next, two specific primers were used to amplify the ligation product. PCR products were then denatured by heat and the single strand DNA was cyclized by splint oligo and DNA ligase. After that, 50 bp single-end sequencing was performed on the prepared library by BGISEQ-500. At least 20 M clean reads of sequencing depth were obtained for each sample. All RNA-Seq raw data were uploaded in Gene Expression Omnibus (GEO ID: GSE105135).

### Analysis of RNA sequencing data

RNA-Seq data were initially filtered to obtain the clean data, including removing reads with adaptors, reads with more than 10% unknown bases or low quality reads (the percentage of low quality bases is over 50% in read). Clean data were aligned to the mouse genome (MM10) using HISAT2 with default parameters^49^. Htseq was used to calculate the raw counts for each sample^50^. Stringtie was used to estimate the expression level of the detected genes as FPKM values^51^. EdgeR was used to evaluate the statistical significance of DEGs and the additive linear model was utilized to compensate the batch effect^52^.

### Differentially expressed gene calculation

Sixteen groups from 65 mice were designed in this study, namely CD (control diet group), DEP2 (day 2 of microglial depletion), DEP5, DEP7, DEP10, DEP14, DEP21, REPOP3 (day 3 of microglial repopulation), REPOP5, REPOP7, REPOP10, REPOP14, REPOP21, REPOP60, LPSCD (LPS challenged normal retina), LPSREPOP (LPS challenged microglial repopulated retina). DEGs of each experimental group were detected compared to the control group. To ensure the reliable and robust analysis, genes detected in at least half of samples size and in at least one of the comparing groups were taken into consideration for DEG calculations. EdgeR was applied to detect DEGs between the compared groups^52^. DEGs were defined as genes with false discovery rate (FDR) less than 0.01 and log_2_ fold change greater than 1(up-regulation) or smaller than −1 (down-regulation).

### Statistical analysis

Results were presented as mean ± standard deviation (SD). One-way analysis of variance with Bonferroni’s *post hoc* was used for multiple comparisons. Independent *t*-test was performed to compare differences between two groups. The statistical significance was defined as *p* < 0.05. All statistics were performed by Prism (GraphPad), SPSS (IBM) or Excel (Microsoft).

## Electronic supplementary material

Supplementary Information
