# Supplementary material for: Dual extra-retinal origins of microglia in the model of retinal microglia repopulation
Source: Cell Discov. 2018 Feb 27;4:9. doi: 10.1038/s41421-018-0011-8 (PMC5827656; doi:10.1038/s41421-018-0011-8)
Supplement: Supplementary file 1 — Supplementary Information [file 41421_2018_11_MOESM1_ESM.pdf]

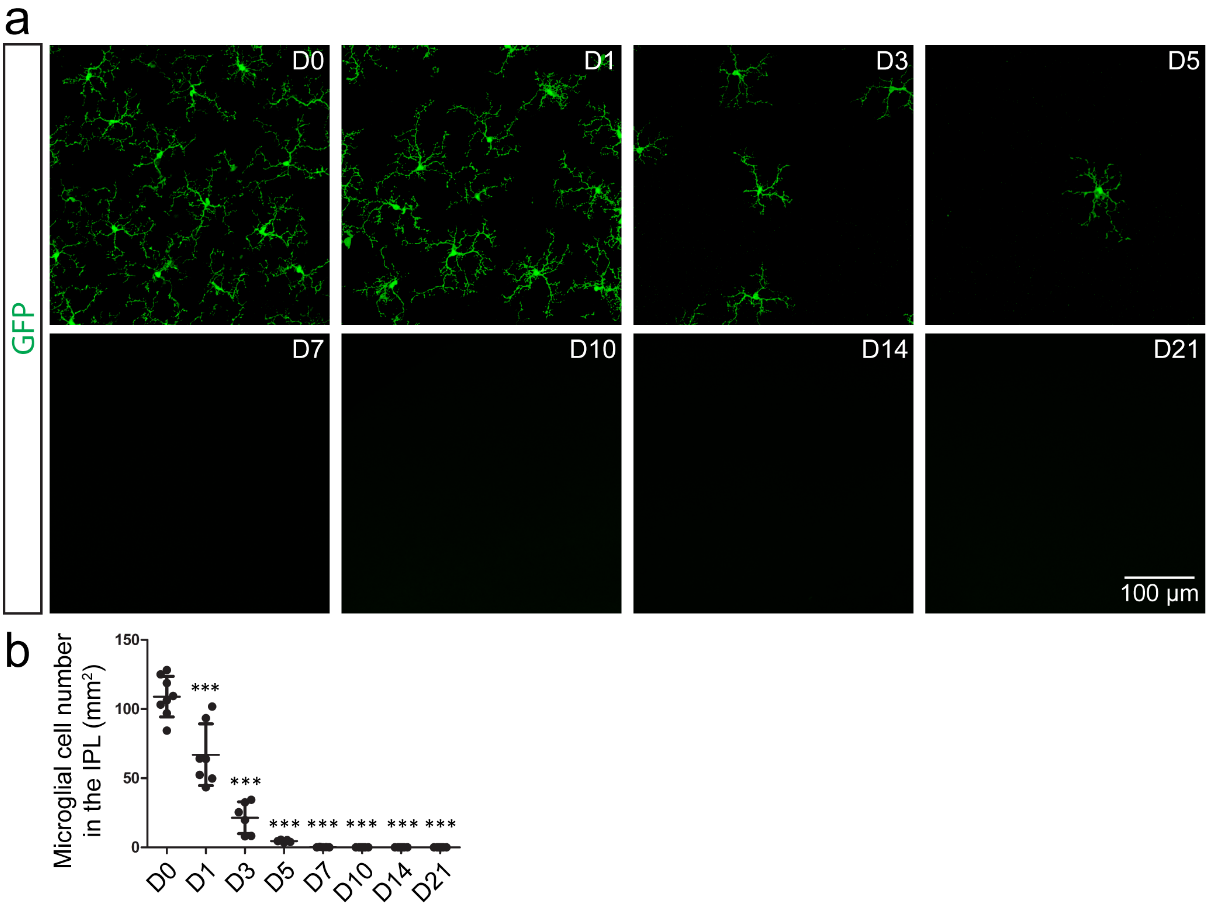

Supplementary Figure 1

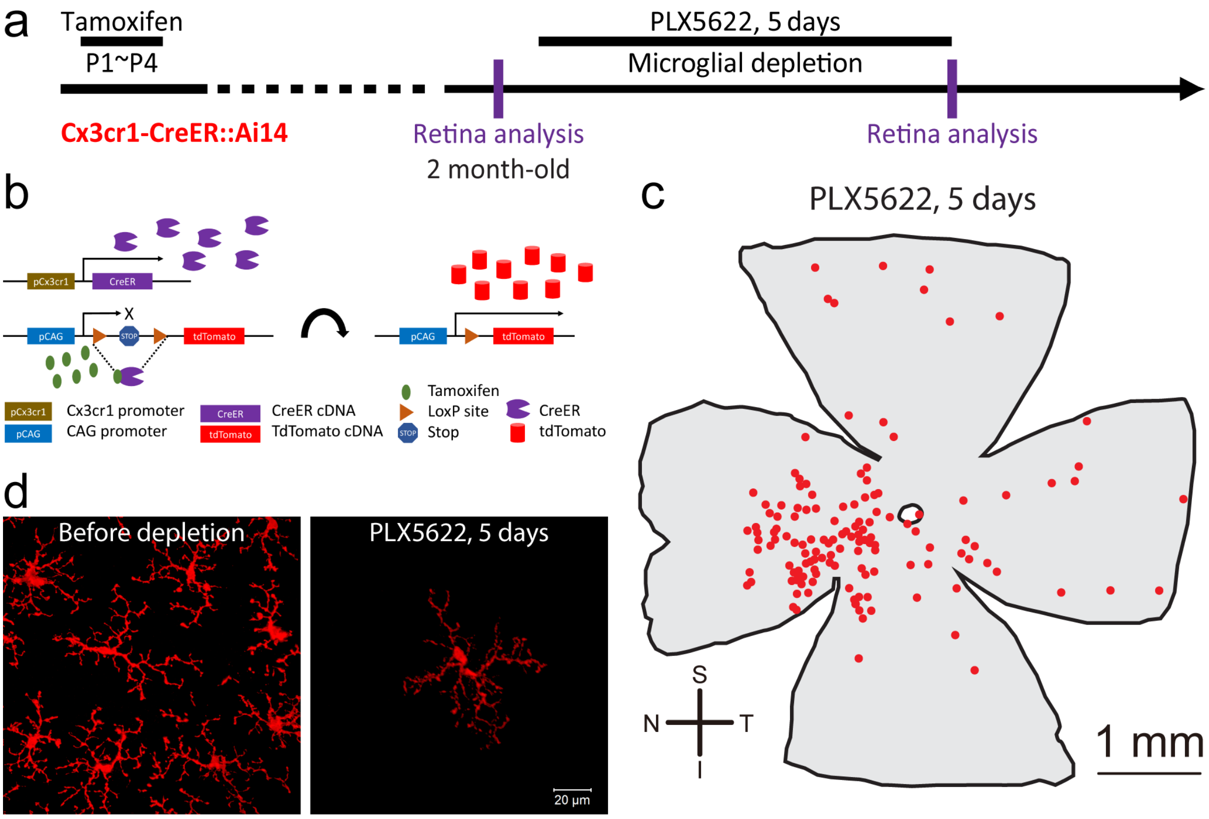

Supplementary Figure 2

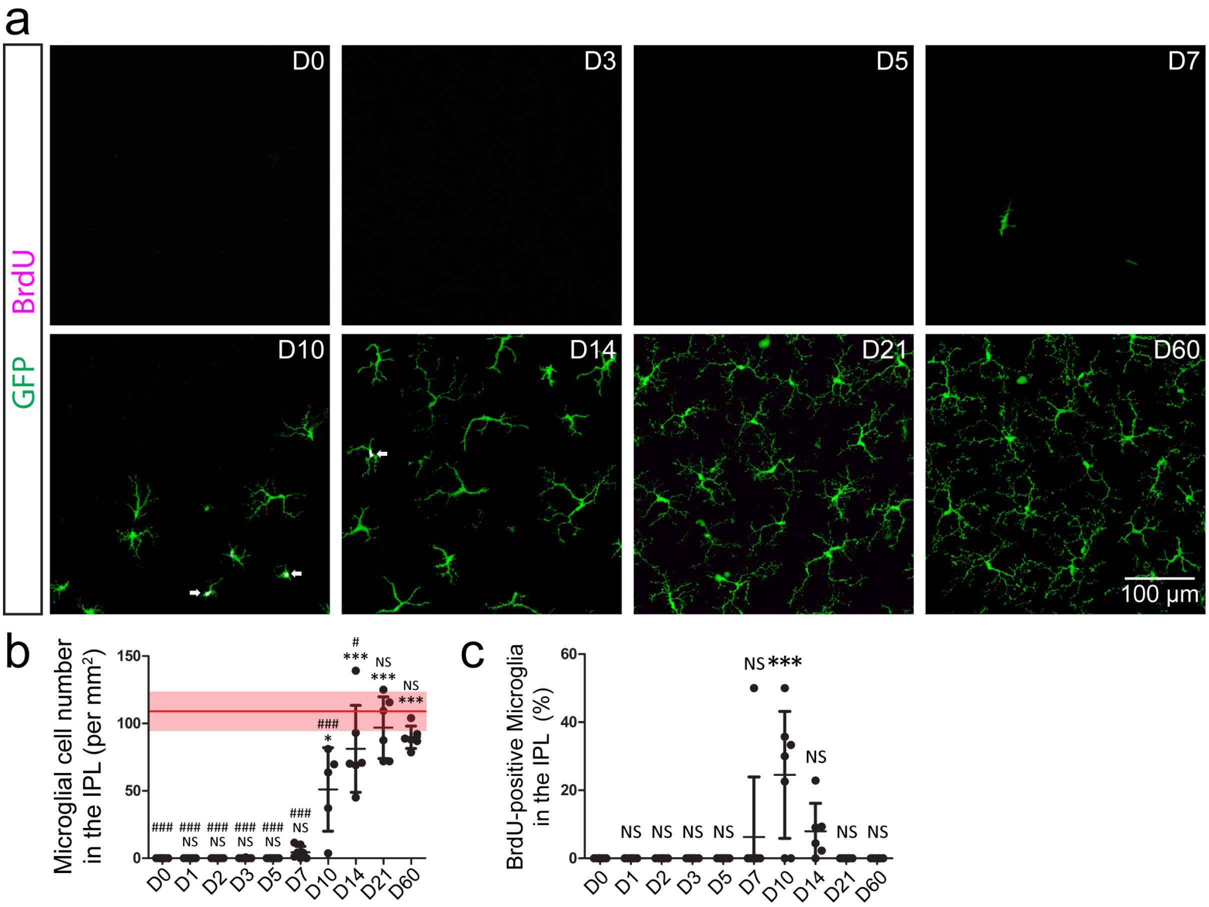

Supplementary Figure 3

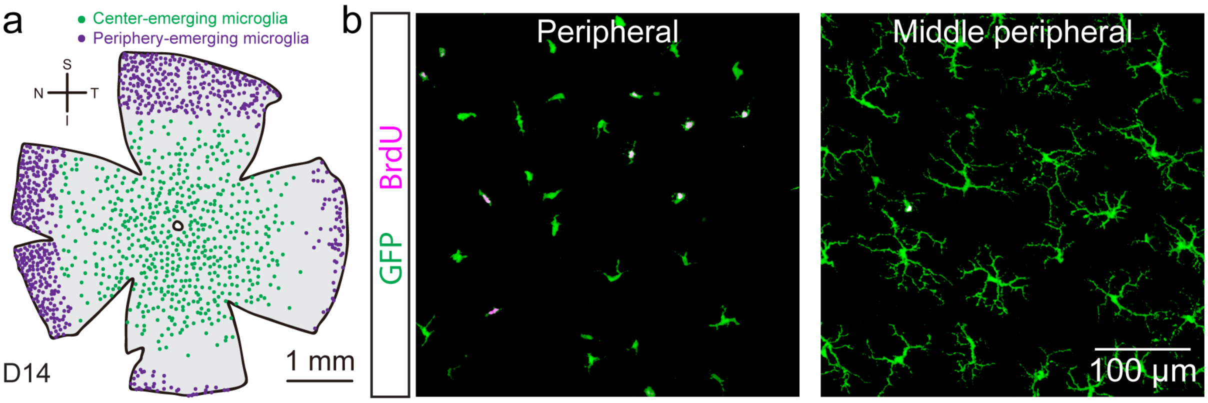

Supplementary Figure 4

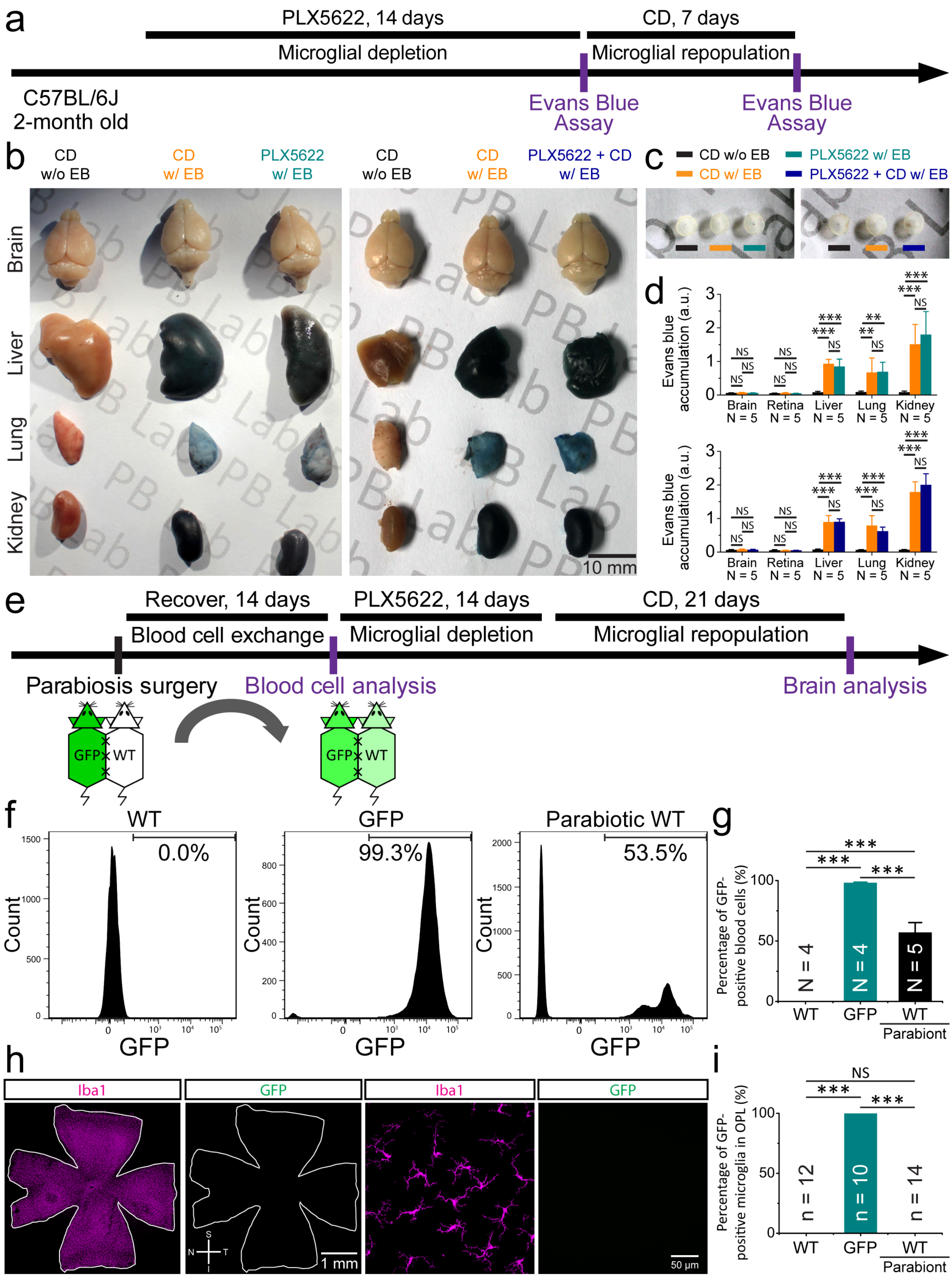

1

2 **Supplementary Figure 5**

3

4

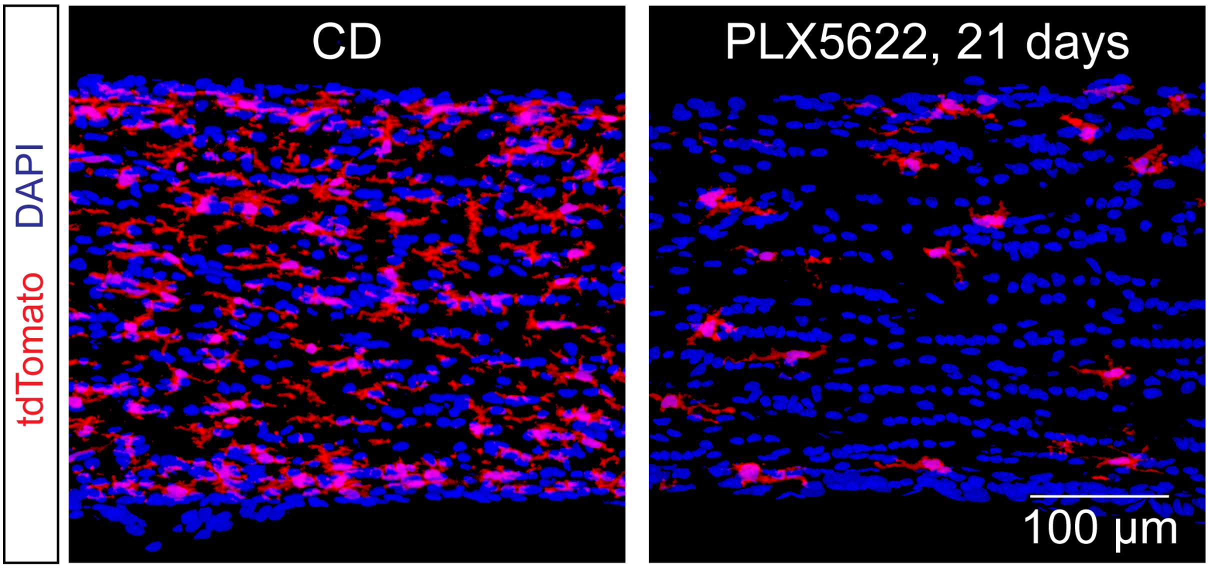

Supplementary Figure 6

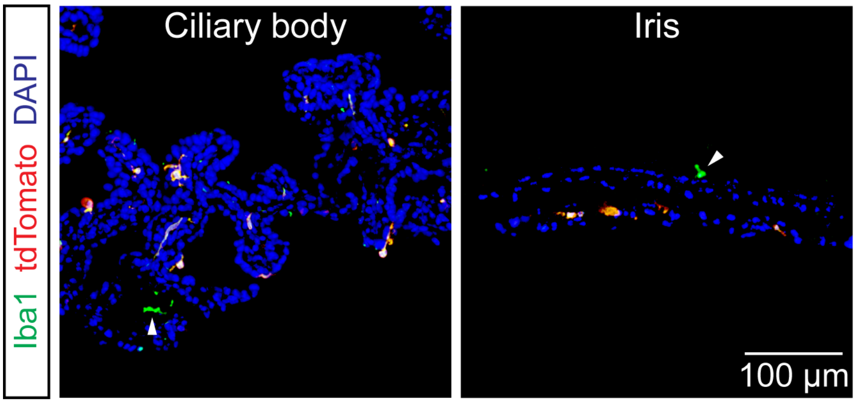

Supplementary Figure 7

Supplementary Figure Legends

Supplementary Figure 1 Inhibition of CSF1R by PLX5622 completely depletes microglia in IPL of the retina.

(a) Representative confocal images show microglial numbers are declined in the IPL of the retina after PLX5622 administration.

(b) Quantification of microglial density in the IPL of normal retinas and the retinas upon PLX5622 treatment.

S: superior; I: inferior; N: nasal; T: temporal; PLX5622: PLX5622 formulated diet. Green: GFP. The data are presented as mean ± SD; NS: not significant; *: $p < 0.05$ to D0; **: $p < 0.01$ to D0; ***: $p < 0.001$ to D0. One-way ANOVA with Bonferroni's *post hoc*.

Supplementary Figure 2 Retinal microglia are indeed depleted upon PLX5622 treatment.

(a) Scheme of genetic inducible fate mapping for resident retinal microglia.

(b) The rationale of tamoxifen-triggered fate mapping.

(c) The spatial distribution of retinal microglia shows that the cell number is largely reduced after PLX5622 administration. Each red dot represents a tdTomato-positive cell.

(d) Representative confocal images show that tdTomato-positive cell number is reduced in the retina after PLX5622 administration for 5 days.

S: superior; I: inferior; N: nasal; T: temporal; PLX5622: PLX5622 formulated diet. Red: tdTomato.

Supplementary Figure 3 Repopulated microglia replenish the IPL after removal of CSF1R inhibition.

(a) Representative confocal images show microglia emerge in IPL after removal of PLX5622.

(b) Quantification of microglial density in the IPL of the retina during microglial repopulation. The red line and pink area indicate the mean and SD of microglial density in the normal retina, respectively. NS: not significant; *: $p < 0.05$ to D0; **: $p < 0.01$ to D0; ***: $p < 0.001$ to D0; #: $p < 0.05$ to the normal retina; ##: $p < 0.01$ to the normal retina; ###: $p < 0.001$ to the normal retina.

(c) Quantification of BrdU-positive microglia in the IPL during repopulation. NS: not significant; *: $p < 0.05$ to D0; **: $p < 0.01$ to D0; ***: $p < 0.001$ to D0.

PLX5622: PLX5622 formulated diet; CD: control diet. Green: GFP; magenta: BrdU.

The data are presented as mean ± SD. One-way ANOVA with Bonferroni's *post hoc*.

Supplementary Figure 4 Two distinct populations of repopulated microglia in the retina.

(a) The representive spatial distribution of retinal microglia shows two distinct groups of microglia in the retina of repopulation for 10 days. Each green dot represents a center-emerging repopulated microglial cell, while each purple dot represents a periphery-emerging microglial cell.

(b) The periphery-emerging microglia in the peripheral retina exhibit distinct morphologies from center-emerging microglia in the middle peripheral retina.

S: superior; I: inferior; N: nasal; T: temporal. Green: GFP; magenta: BrdU.

Supplementary Figure 5 Repopulated microglia in the retina are not derived from blood cells.

(a) Scheme and time points of Evans blue assay for the BRB/BBB integrity examination.

(b-c) Brains, peripheral organs (b) and retinas (c) from the microglia-depleted and microglia-repopulated mice 6 hours after Evans blue administration.

(d) Quantifications of the Evans blue intensity in retinas, brains and peripheral organs of microglia-depleted and microglia-repopulated mice.

(e) Scheme for testing the blood cell-derived pathway by parabiosis.

(f-g) Percentages of GFP-positive blood cells in the WT, beta-actin-GFP and WT parabiotic mice.

(h) Confocal images show that there are no GFP-positive repopulated microglia in the retina of WT parabionts.

(i) Quantifications of GFP-positive microglia in the OPL of the retinas in the WT, $Cx3cr1^{+/GFP}$ and parabiotic WT mice.

PLX5622: PLX5622 formulated diet; CD: control diet. Green: GFP; magenta: Iba1 (microglial marker). The data are presented as mean ± SD. N: mouse number for each group; n: retina number for each group; NS: not significant; *: $p < 0.05$; **: $p < 0.01$; ***: $p < 0.001$. One-way ANOVA with Bonferroni's *post hoc*.

Supplementary Figure 6 Microglia in the optic nerve are labeled by tdTomato in tamoxifen-treated Cx3cr1-CreER::Ai14 mice.

Confocal images show that PLX5622 treatment for 21 days does not deplete all microglia in the optic nerve.

Red: tdTomato; blue: DAPI.

Supplementary Figure 7 Some macrophages in the ciliary body and iris are not labeled by tdTomato two months after tamoxifen injection.

Confocal images show that some macrophages in the ciliary body and iris are not labeled by tdTomato two months after tamoxifen injection.

Red: tdTomato; cyan: Iba1; green: Iba1; blue: DAPI. Arrow heads: tdTomato-negative macrophages.
